# DIY enzyme labelled fluorescence alcohol (ELFA) standard production protocol to quantify single-cell phosphatase activity (SCPA) of microplankton

**DOI:** 10.1016/j.heliyon.2020.e05582

**Published:** 2020-11-26

**Authors:** Daniel Diaz-de-Quijano, Cleo N. Stratman, Stella A. Berger

**Affiliations:** a Siberian Federal University, Fac. of Fundamental Biology and Biotechnology, Dept. of Aquatic and Terrestrial Ecosystems, Svobodnyy Prospect 79, 660041, Krasnoyarsk, Territory of Krasnoyarsk, Russian Federation; b Leibniz-Institute of Freshwater Ecology and Inland Fisheries (IGB), Department of Experimental Limnology, Alte Fischerhütte 2, D-16775 Stechlin, Germany

**Keywords:** Enzyme labelled fluorescence (ELF), Fluorescence labelled enzyme activity (FLEA), ELF®-97 phosphate (ELFP), Single-cell phosphatase activity (SCPA), Phosphatase activity, Phytoplankton, Environmental enzyme activity, Algal biology, Freshwater ecology, Microorganism, Enzyme kinetics, Enzymology, Nutrient availability

## Abstract

Extracellular enzyme activities (EEA) are crucial components of microbial food web interactions and biogeochemical cycles in aquatic ecosystems. They also represent relevant biological traits in the ecophysiology of phytoplankton and other components of microbial plankton. To assess species-specific and (sub-)population-level characteristics of phytoplankton EEA at the single-cell level and close-to-*in-situ* conditions solely the enzyme labelled fluorescence (ELF)-based substrates have been used, because they become fluorescent and precipitate around the enzyme activity location upon enzymatic cleavage. However, the enzyme-labelled fluorescence alcohol (ELFA) standard is no longer commercially available, hence standard curves cannot be run anymore and single-cell phosphatase activity (SCPA) is no longer quantifiable. Therefore, we introduce a simple protocol for an ELFA standard do it yourself (DIY) production to enable quantifying microplankton SCPA again. This protocol is based on fluorescence measurements easily available to environmental enzyme activity laboratories, and it circumvents any need for chemical synthesis equipment and knowledge. The method is based on a controlled reaction of the ELF-phosphate (ELFP) substrate with commercially available alkaline phosphatase, which efficiently turns all the substrate into ELFA product. The ELFA product was dried out and dissolved again in dimethyl sulfoxide (DMSO) for storage. The ELFA concentration of that standard-to-be ELFA solution in DMSO was determined by linear regression between a low concentration dilution series of ELFA solution measured fluorimetrically and parallel measurements of a series of phosphatase-catalysed reactions at an overlapping ELFP concentration range. Finally, the fluorescence- and concentration-stable ELFA solution in DMSO with a known concentration constitutes the ELFA standard that is necessary to quantify bulk (fluorimeter) and single-cell (microscope and flow cytometer) phosphatase activity in microplankton.

## 1 Introduction

Enzyme activities are the means by which microorganisms accelerate chemical reactions within and around them to match the tempo of their biological demands of matter and energy. Carbon and nitrogen fixation, hydrolysis of organic molecules in the environment to make them bioavailable and redox transformation of inorganic nitrogen species are examples of microbial enzyme-catalysed reactions with an impact on biogeochemical cycles. The study of enzyme activities in the environment is likely to continue in the post-omics era, as enzyme activities are the missing link between the three steps of the central dogma of biology, i.e. gene presence, its expression and protein content, and the actual rates of nutrient cycling. Moreover, enzyme activities play a crucial role in microbial food web interactions by linking the organic matter size continuum to different microbes. With bacteria accounting for a big share of enzyme activities in the environment, phytoplankton has been identified as the main contributor to phosphatase activity in many aquatic ecosystems (e.g. Refs. [1, 2]). Phosphatase activity has largely been used as an indicator of phosphorus limitation for phytoplankton growth in lakes and coastal oceans [3, 4, 5, 6, 7]. Enzyme kinetics and specifically phosphatase activity is also an important phytoplankton trait that might be worth to be included in the upcoming phytoplankton species biological traits databases [8, 9].

An especially valuable essay in that field has been the enzyme-labelled fluorescence (ELF) technique for single-cell phosphatase activity (SCPA). It is based on the phosphatase-catalysed hydrolysis of the phosphomonoester bound in the ELF-phosphate substrate (ELFP). The resulting ELF-alcohol product (ELFA) becomes fluorescent and precipitates around the enzyme location. The ELFP substrate is a modification of the ELFA fluorochrome, which was synthesised for histological and cytological purposes [10, 11, 12, 13, 14], and has been successfully used on aquatic microbes for two decades [15], still holding new potentialities to be explored.

The most evident contribution of this single-cell culture-free technique was the determination of identity-to-function relationships within natural phytoplankton communities. The identity of phosphatase active species in environmental samples includes many representatives within chlorophytes, diatoms, chrysophytes, dinoflagellates, euglenophytes, cryptophytes and cyanobacteria (e.g. Ref. [16]). One of the strengths of the ELF technique is the wide taxonomic range it can be applied to, including other actors of the microbial community such as heterotrophic bacteria [17, 18, 19, 20, 21, 22], heterotrophic flagellates [23, 24, 25] and rotifers [26]. Additionally, within any particular phytoplankton community, only some species or genera have shown to be phosphatase-active and, likewise, not even all individuals within a population turned out to actually be active [27, 28]. The fact that ELF is a single-cell technique makes it suitable to inform about the potential role of intra-population and intra-community variability on ecosystem level processes.

The second major asset of the ELF technique is its quantitative application, i.e. the quantity of the enzyme reaction product can be measured. Fluorimetric enzyme activity assays like ELF are more sensitive than colorimetric assays, allowing to use the whole range of low to high substrate concentrations. This includes for example low close-to-*in-situ* substrate concentrations in assays as well as high concentrations needed for enzyme saturation in enzyme kinetic approaches. ELFA is an excellent candidate for quantification of enzyme activities not only because it is fluorescent, but also because it is a bright and stable fluorochrome with excitation and emission spectra (ex. 350 nm, em. 530 nm) clearly distinguishable from the background biological specimens. Its fluorescence can be measured using flow cytometry, epifluorescence and confocal microscopy [29, 30, 31, 32, 33]. Furthermore, a protocol to convert relative fluorescence units (RFU) into actual rates of phosphatase-hydrolysed ELFA or phosphorus has been published [34]. The latter step is crucial because it converts quantitative continuous ELF-SCPA fluorescence into biogeochemically meaningful and comparable units. Besides enzymatic rate measurements, ELF-based SCPA quantification can be used to separately assess enzyme kinetics of particulate environmental microalgae populations at close-to-*in-situ* conditions (Diaz-de-Quijano *et al.*, in prep.).

Future potentialities of the ELF technique arise from the fact that it is a non-destructive technique. Because the integrity of ELF treated cells is preserved we can identify them and combine ELF with other single-cell probes and measurements. Some potential examples are the combination of ELF with microautoradiography, stable isotope probing (SIP) and NanoSIMS, fluorescently labelled bacteria (FLB), and emerging single-cell enzyme activity (SCEA) substrates. Combining ELF with microautoradiography or SIP and NanoSIMS would allow to assess the link between hydrolytic enzyme activity and nutrient uptake, which are biogeochemical processes that occur at the cell level. Further, shifts in phosphorus scavenging strategies in mixotrophic phytoplankton such as phosphatase activity and bacterivory can be assessed via a combination of ELF and FLB. Finally, single-cell multi-enzymatic assays can be developed by combining the ELFP substrate with selected enzyme substrates from the range of available products [35].

ELF-based substrates for enzyme activities other than phosphatase are unfortunately not commercially available anymore. Three more assays used to be available in the past: ELF-glucuronide for β-glucuronidases [36, 37], ELF-N-acetly-β-D-glucosaminide for β-N-acetyl-hexosaminidases, chitinases and/or lysozymes [26, 38, 39, 40], and ELF-palmiate for lipases [26]. At that time, the ELF technique used to be called fluorescently labelled enzyme activity (FLEA) for syntactic and semantic reasons but also because of this span of ELF substrates [41]. Indeed, these ELF-based substrates for different enzyme activities were not simultaneously combinable because they all produced the same ELFA molecule with identical fluorescence characteristics. Recently synthesised precipitating [42] and covalent binding [43] alternative substrates for phosphatase [and sialidase, [44]] will contribute to achieve simultaneous single-cell multi-enzymatic assays. Widening the range of enzyme substrates and excitation/emission spectra of the reaction products is the basic prerequisite to make them happen. Finally, the non-destructive quality of the ELF technique and the knowledge on different ELF protocols makes it suitable for the toolbox of *in vivo* techniques in microbiology [45, 46].

Many of the attained and potential achievements of the ELF technique are based on the fact that the product of SCEA-catalysed reactions could be quantified. To quantify that, it is imperative to use an ELFA standard, i.e., an ELFA solution with known and stable ELFA concentration and fluorescence [34]. However, the ELFA standard (1 mM) production has been discontinued by Thermo Fisher Scientific, the parent company that owns Invitrogen, which in turn had previously purchased Molecular Probes, and only ELFP is still commercially available. Self-production of the ELFA standard and different ELF-based enzyme substrates would in principle be feasible following the described protocols, but the required reagents, laboratory equipment and knowledge is usually out of reach for aquatic sciences end users [18, 37, 38]. Here, we suggest a simple DIY-protocol to obtain an ELFA standard solution using fluorimetric methods available to any aquatic ecology laboratory with basic enzyme activity equipment. Succinctly, we react ELFP substrate with phosphatase, dry resulting ELFA, dissolve it again in DMSO and determine its ELFA concentration. We followed two strategies to determine the final ELFA concentration: (1) by sequentially diluting a concentrated ELFA solution and checking for the fluorescence slope break at 1 mM ELFA as described in the literature [10], and (2) by determining the reaction efficiency under identical conditions to the ELFP reaction but using a different substrate (4-methylumbelliferyl phosphate, MUFP) that cannot be used at the single-cell level but whose standard (4-methylumbelliferone, MUF) is commercially available. We discuss the ins and outs of each step and conclude with a protocol, which will allow continuing to use the ELF technique quantitatively.

## 2 Materials & methods

### 2.1 Enzyme reactions

Different reactions of ELFP (Thermo Fisher Scientific, E6589) and MUFP (Sigma, M8883) substrates were run in the dark at +37 °C using 254.5 units·ml^−1^ alkaline phosphatase (EC 3.1.3.1, Sigma, P6774), 0.01 M Tris buffer pH 7.9, and 10 mM MgCl_2_ final concentrations. Firstly, single-point high concentration (1.5 mM) ELFP and MUFP reactions were run in triplicate and quadruplicate, respectively. Duplicate MUF (Aldrich, M1381) calibration curves were measured in parallel. Secondly, triplicate MUFP reactions and MUF calibration curves were compared using a concentration series several orders of magnitude lower than in the former case (1, 0.8, 0.6, 0.4, 0.2, 0.1, 0.05, 0.025, and 0 μM). Finally, triplicate ELFP reactions were run in the same low concentrations. We ran reactions at pH 7.9 because it is within the alkaline phosphatase stability range (pH 7.5–9.5) and it is safely below the threshold of pH 8 above which phosphatases might hydrolyse ELFP into ELFA but precipitation and fluorescence of ELFA is importantly reduced by phenolic ionization [11]. This way, we could monitor ELFA fluorescence increase from the lowest concentrations until the plateau (1 mM, Huang et al. 1992). That was especially important for reactions with 1.5 mM ELFP, which were intentionally run at a concentration safely above the ELFA fluorescence plateau. Those reactions were monitored until the plateau was reached and then they were let to react further on, long enough so that all the substrate had time to turn into product.

### 2.2 ELFA drying and dissolution

The aqueous ELFA solutions resulting from the three 1.5 mM ELFP reactions were dried out in two different ways. To accelerate the drying step, the ELFA solutions were amended with either 4:3 ELFA solution:95% acetone or 3:2 ELFA solution:70% ethanol (v:v), kept in the dark, protected from dust, and let evaporate in the heating oven at +37 °C over night. Finally, the dry ELFA crystals were dissolved back into 200 μl DMSO, pipetting up and down more than 100 times to the different cardinal directions.

### 2.3 Instrumentation

A BioTek Synergy 2 microplate reader (BioTek Instruments, Inc., Bad Friedrichshall, Germany) was used to read ELFA and MUF fluorescence in black microplates at ex. 360/40 nm and em. 528/20 nm (ELFA) or em. 440/40 nm (MUF). Sensitivity 35 was used for 1.5 mM substrate reactions, whereas sensitivities 80 and 60 were used to measure ELFP and MUFP reactions up to 1 μM initial substrate concentration, respectively. Previous commercial ELFA calibration curves are also presented for comparison with current measurements. They were measured in a 1-cm quartz cuvette using a Shimadzu RF-5301 PC spectrofluorometer equipped with a xenon lamp, at ex. 350/30 nm and em. 530/20 nm.

### 2.4 Statistics

Different libraries (segmented, built-in and lsmeans) in the R statistical environment were used to find breakpoints in MUF dilution series, to calculate and compare least-squares regression scores and test for differences between them [47].

## 3 Results and discussion

### 3.1 High concentration reactions

**Figure 1:**
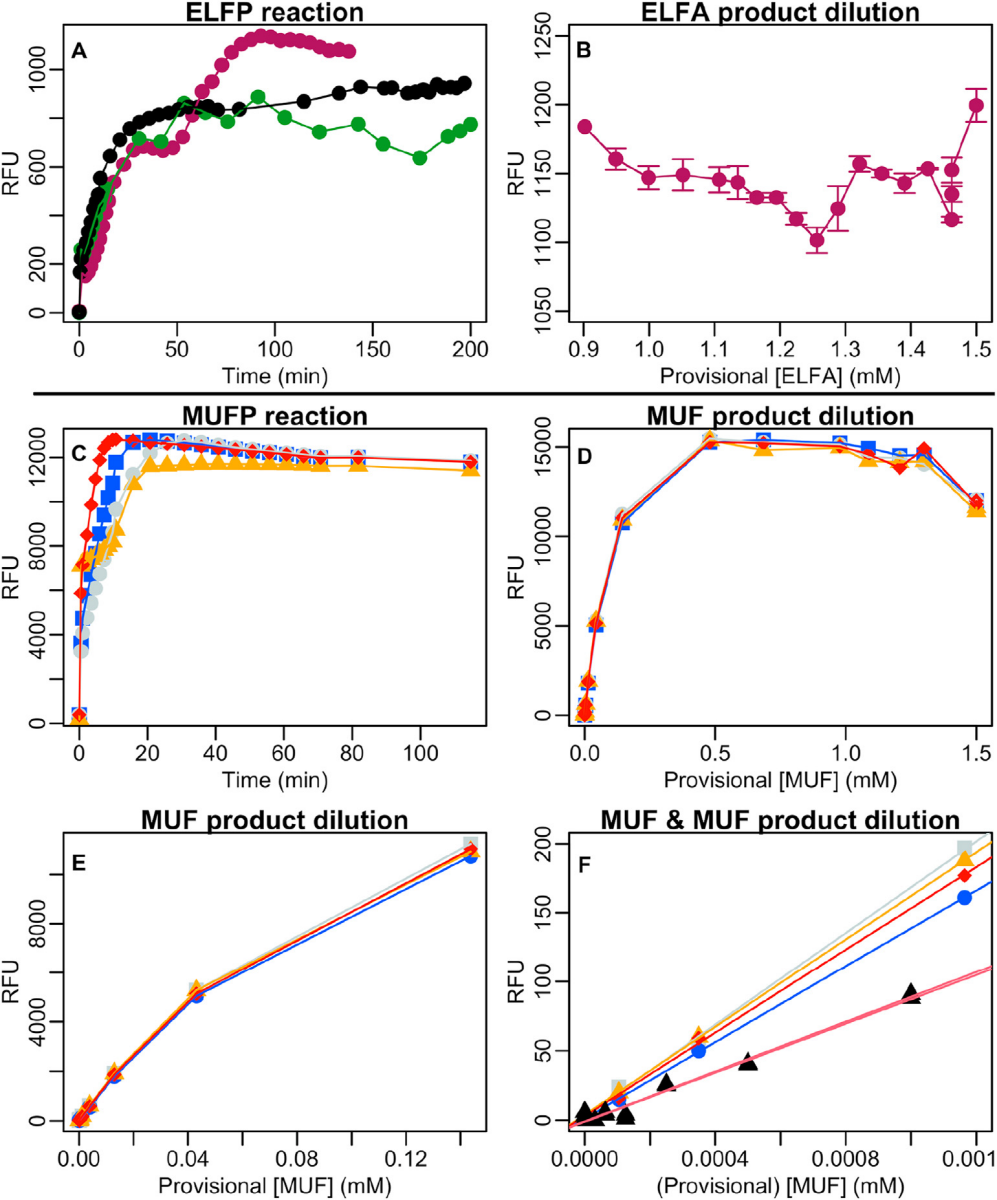
A: Time course of replicate alkaline phosphatase reactions with 1.5 mM ELFP (enzyme labelled fluorescence phosphate) substrate, in relative fluorescence units (RFU). ELFP reaction replicates in black, green and purple. B: Dilution series of one of the resulting ELFA (enzyme labelled fluorescence alcohol) solutions. C: Time course of replicate alkaline phosphatase reactions with 1.5 mM MUFP (4-methylumbelliferyl phosphate) substrate under the same conditions as the ELFP reaction (A). MUFP reaction replicates in light grey, yellow, blue and red. D, E and F: Different concentration ranges of the dilution series of the MUF (4-methylumbelliferone) solutions obtained from the reaction in C, and comparison to commercial MUF dilution series (black triangles and red line, F). Provisional fluorochrome concentrations were attributed assuming the ideal case where initial substrate concentration turned into identical product concentration, without any additional loss of fluorescence or fluorochrome. Data shown in Figure 1 is available at Table S1.

Phosphatase-catalysed hydrolysis reactions were run under identical conditions for 1.5 mM ELFP and MUFP substrates, and monitored over time fluorimetrically (Figure 1 A and C). After that, sequential dilutions of the resulting MUF and ELFA solutions were attempted but two problems in the ELFA case arose. Firstly, final volume was not 200 μl but lower. Evaporation during enzyme reaction at +37 °C depended on reaction time and sample handling, resulting in different final volumes of the replicates (144, 169 and 161 μl). Secondly, the resulting ELFA aqueous solutions at pH 7.9 contained large ELFA crystal aggregates, visible by naked eye. We decided to run the reaction at pH 7.9, below 8, intentionally to allow ELFA precipitates to form and fluoresce (see section 2.1), monitor the reaction time-course and let the reaction run enough time to make sure all the ELFP substrate had enough time to turn into ELFA product. Nevertheless, crystal size in the final solution hampered any accurate dilution or manipulation. Some ELFA traces stayed even on the bottom of the initial microwell, when transferring the volume from one microwell to another. Therefore, we decided to dry out the ELFA solution, dissolve it again in DMSO, and then perform the dilution series.

Because the evaporation step was especially slow, we used different dry down solvents to speed it up. Acetone (boiling point 56 °C) was tested in the first ELFA replicate, whereas ethanol (b.p. 78.4 °C) was used in the other two replicates, following ELFP manufacturers suggestion (see section 2.2). Both solvents successfully speeded up the evaporation step. The following step, dissolution of dry ELFA crystals into 200 μl DMSO was a challenging step, as a few crystals still remained attached to the microwell bottom (first and second replicates). The evaporation and dissolution steps were improved when manipulating the third replicate. The original third replicate 161 μl ELFA aqueous solution was mixed with 100 μl 70% ethanol, pipetting up and down. As a result, ELFA crystals dissolved. A 70 μl DMSO layer was added with the pipette tip directly on the bottom of the microwell. In this way, ELFA crystals that would form during the upper layer evaporation were settled down and dissolved into the non-evaporating DMSO layer (b.p. 189 °C). Finally, 130 μl DMSO were added next day to achieve 200 μl volume. DMSO is an ideal solvent to keep ELFA concentration stable in time (for at least 1–2 years) because its high boiling point minimises any further evaporation. DMSO has already been used in the former commercially available ELFA standard.

ELFA solutions in DMSO were sequentially dissolved below the 1 mM threshold where fluorescence was expected to decrease, but it did not (Figure 1 B). The reason is that the ELFA fluorescence plateau described in the literature was observed with an ELFA solution in water [10], but not in DMSO. In an aqueous solution, the ELFA molecule forms an internal hydrogen bond that dramatically increases its fluorescence and makes it precipitate, which does not occur in DMSO. Although our dilution series was performed by adding water on the ELFA solution in DMSO, the water content was too low for ELFA to become fluorescent at its maximum yield. Fluorimeter sensitivity had to be increased from 35 to 60 to monitor the dilution series of mostly hydrogen bound-lacking non-fluorescent ELFA molecules but, obviously, the characteristic fluorescence plateau was not observable under that molecular conformation. The alternative explanation to the fact that we did not observe a decline of the fluorescence slope was that the dilution range had not actually crossed the 1 mM threshold. However, that hypothesis was discarded. The dilution range clearly crossed the 1 mM threshold because the starting point of provisional 1.5 mM ELFA is an arbitrary value set as a maximum reference to guide the dilution process. The actual concentration was below this provisional value because some ELFA crystals were lost during dissolution in DMSO and manipulation. Initially, we also considered the possibility that some percentage of ELFP substrate eventually had not turned into ELFA product, although we later considered this option to be very unlikely, based on successful MUFP reactions.

The MUF product is soluble itself, thus no intermediate drying and dissolving in DMSO steps were needed, and it was directly diluted and measured. Saturating fluorescence was observed above provisional 0.5 mM MUF (Figure 1 D). Fluorescence to MUF concentration relationship was non-linear from provisional 0.031 to 0.22–0.5 mM (Figure 1 D and E), and linear below provisional 0.031 ± 0.003 mM MUF (see also Figure 1 F), indicated by the breakpoint analysis. To assess the efficiency of the phosphatase-catalysed hydrolysis of MUFP into MUF we compared the linear segment of the dilution series to a calibration line based on commercially available MUF (Figure 1F, black triangles and red line). In the case where the phosphatase-catalysed hydrolysis of MUFP into MUF had been 100% efficient, the resulting provisional MUF concentration should perfectly match both the initial MUFP concentrations and the commercially available MUF calibration line. Alternatively, a reaction efficiency below 100% would trigger a reacted MUF line below that of the commercial MUF. Surprisingly, none of those was the case. The linear part of the hydrolysed MUF product had a significantly steeper regression of means slope than commercial MUF (F (1,7) = 22.4, p-value = 0.0021). According to this unexpected result, we could not determine the reaction efficiency to be 100% or lower than 100%. This could be due to empirical errors such as commercial MUF underweighing, MUFP overweighing, and/or inaccurate restoration of hydrolysed MUF solution back to 200 μl. In any case, the linear part of the hydrolysed and commercial MUF regression lines (Figure 1 F) were based on only 3 and 5 concentrations, respectively, which covered only partially overlapping ranges.

### 3.2 Low concentration MUFP reactions

**Figure 2:**
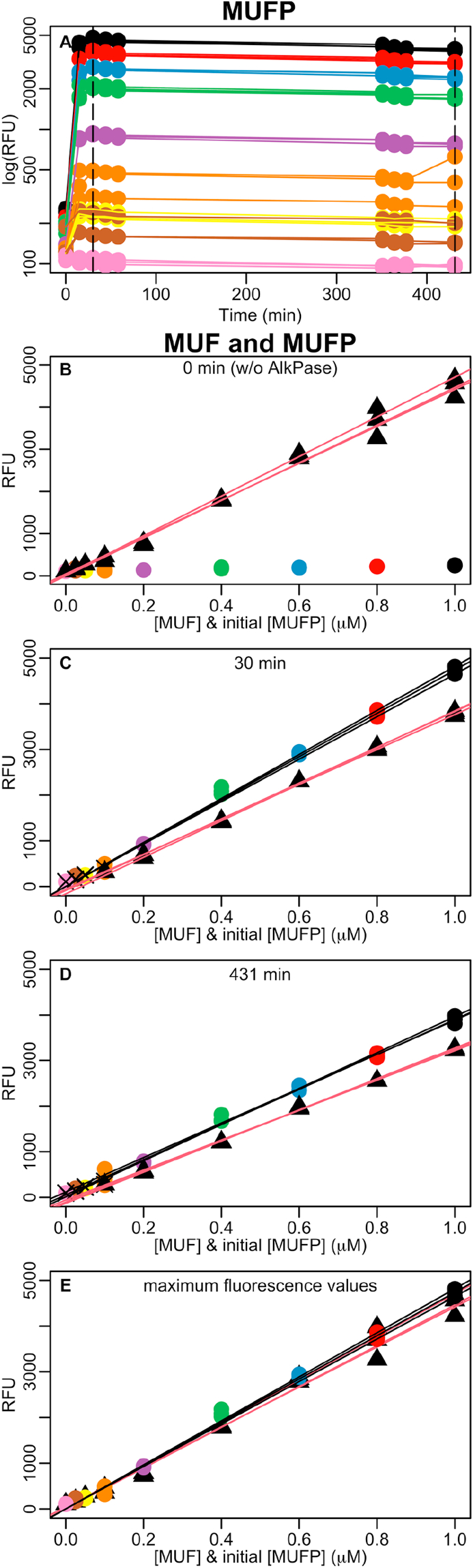
A: Time courses of alkaline phosphatase reactions with different triplicate MUFP substrate concentrations: 1 μM (black), 0.8 μM (red), 0.6 μM (blue), 0.4 μM (green), 0.2 μM (purple), 0.1 μM (orange), 0.05 μM (yellow), 0.025 μM (brown), and 0 μM (pink). Dashed vertical lines show selected time points (30 and 431 min). B, C and D: comparison of triplicate reactions (coloured circles and black lines) and triplicate commercial MUF dilution series (black triangles and red lines) at different time points: before alkaline phosphatase addition (B), and after 30 (C) and 431 (D) minutes reaction. “X” symbols account for observations not included in the linear regressions because reduced the Rˆ2. E: comparison of the highest recorded fluorescence values (commercial MUF at min 0 and hydrolysed MUF at min 30). Data shown in Figure 2 is available at Table S2.

Multiple MUFP reactions were run at the same conditions as the high concentration reactions but at nine different concentrations safely under the MUF quenching threshold to overcome previous uncertainties (Figure 2 A). Commercial MUF calibration lines at the same concentrations were measured simultaneously to MUFP reactions to avoid any bias due to evaporation or fading. The first half hour after reaction start, hydrolysed MUF already overpassed commercial MUF regression slope (F (1,11) = 57.7, p-value < 0.0001) (Figure 2 B, C, D). These results confirm those observed in the previous section but the possible empirical errors that could have triggered higher reacted than commercial MUF fluorescence there were avoided here. The time course of the reaction is the gist to interpret why fluorescence was higher in reacted than in commercial MUF. Actually, commercial MUF started fading while alkaline phosphatase was being added to the wells with MUFP substrate and while most of the MUFP was still undergoing hydrolysis and transforming into reacted MUF, during the first 30 min of reaction. From then on, both commercial and reacted MUF bleached at a similar pace. That probably occurred because dark conditions were interrupted several times during a long incubation to monitor its progress in the microplate reader. Consequently, comparing maximum fluorescence of commercial MUF (Figure 2 B, black triangles and red lines) and reacted MUF (Figure 2 C, coloured circles and black lines) was the only way to minimise fading interference in results. In so doing, the regression lines of commercial and reacted MUF had not significantly different slopes (F (1,14) = 3.03, p-value = 0.1037) and slightly but significantly higher intercept in reacted as compared to commercial MUF (F(1,14) = 6.32, p-value = 0.0248) (Figure 2 E). In conclusion, the phosphatase-catalysed reaction of MUFP into MUF is very likely to be 100% efficient and, in case it was slightly lower, it would be insignificantly lower as compared with experimental error caused by fluorochrome fading.

### 3.3 Low concentration ELFP reactions

**Figure 3:**
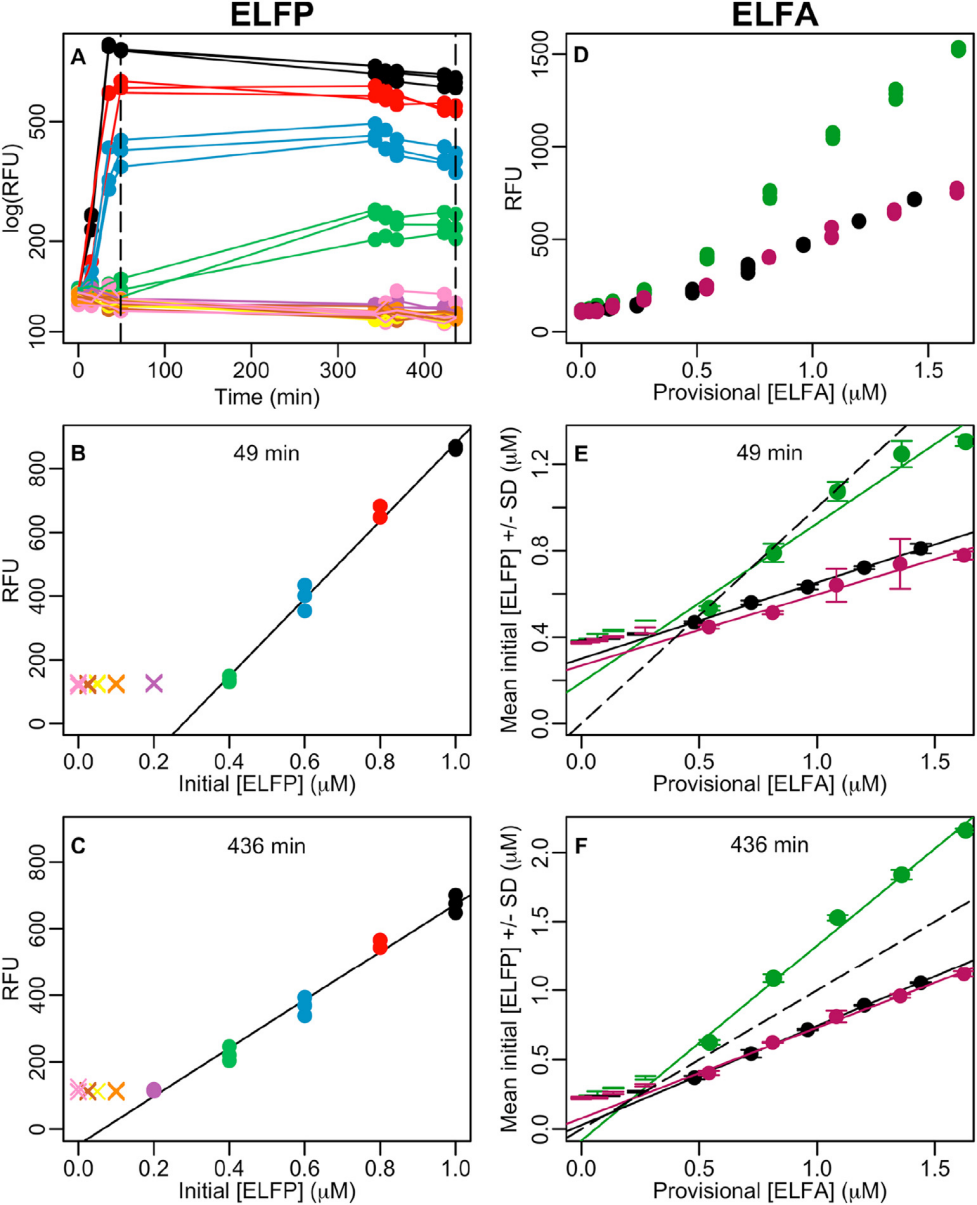
A: Time courses of alkaline phosphatase reactions with different triplicate ELFP substrate concentrations: 1 μM (black), 0.8 μM (red), 0.6 μM (blue), 0.4 μM (green), 0.2 μM (purple), 0.1 μM (orange), 0.05 μM (yellow), 0.025 μM (brown), and 0 μM (pink). Dashed vertical lines show selected time points (49 and 436 min). B and C: relationship between the initial ELFP concentration and fluorescence at two different time points of the time course, and linear regressions of the range with linear relationship. D: fluorescence measurements of ELFA dilution series (ELFA solution 1 black, 2 green, and 3 purple, whose production was monitored in Figure 1 A) at minute 436. E and F: relationship between provisional ELFA concentration of the 3 ELFA solutions dilution series and their actual ELFA concentrations using regression lines of RFU to initial ELFP concentrations (like in B and C but inverted). Dashed lines show 1:1 relationship. Data shown in Figure 3 is available at Table S3.

The very same multiple reactions were run using ELFP as a substrate and monitored along time (Figure 3 A, B, C). The fluorescence to initial ELFP concentration relationship was determined for the linear range at two time points (Figure 3 B and C). Although different substrates might have different kinetic properties, we assumed that all the initial ELFP substrate turned into ELFA product, as observed twice in the case of MUFP. After that, the initial ELFP would equate to hydrolysed ELFA concentration. Simultaneously to the low concentration (≤1 μM) ELFP reactions we also measured a dilution series of the three ELFA solutions previously obtained in section 3.1 from phosphatase-catalysed reactions of 1.5 mM ELFP, evaporation and dissolution in DMSO (Figure 3 D). The high dilution factor we used was comparable to the one used with formerly commercially available 1 mM ELFA standard in DMSO. The dilution was sufficient to avoid any DMSO interference in the formation of the intramolecular hydrogen bound necessary for ELFA to precipitate and fluoresce. Approximate provisional ELFA concentrations were assigned to each of the three standard-to-be ELFA solutions in order to build up dilution series within comparable ranges. These assignments were based on dilutions performed in previous steps (e.g. Figure 1 B), assuming the ideal case where no crystals or fluorescence were lost. Our overall goal was to determine the actual ELFA concentration of these standard-to-be ELFA solutions. The linear parts of the ELFA dilution series (Figure 3 D) were converted into initial ELFP, or hydrolysed ELFA, and plotted against provisional ELFA (Figure 3 E and F). Regression lines in the latter two graphs were used to convert the approximate provisional ELFA concentrations to actual ELFA concentrations in the three standard-to-be ELFA solutions in DMSO (Table 1). These stable solutions in DMSO with known ELFA concentration could be used as ELFA standard solutions.

**Table 1:** Provisional and estimates of actual ELFA concentrations of the three standard-to-be ELFA solutions in DMSO. Regression parameters correspond to lines shown in Figure 3 E and F (min 49 and 436 respectively) and a selection of maximum fluorescence values for each initial ELFP concentration.

ELFA solution ID	provisional [ELFA] (μM)	Min 49			Min 436			Maximum fluorescence values		
		slope	intersect	estimate	slope	intersect	estimate	slope	intersect	estimate
1	**200**	0.35	0.30	**71**	0.72	0.03	**143**	0.48	0.13	**96**
2	**453**	0.74	0.19	**333**	1.41	-0.09	**638**	0.99	-0.02	**449**
3	**902**	0.33	0.27	**296**	0.65	0.08	**588**	0.37	0.19	**336**

Bold refer to the main components in the dilution.

**Figure 4:**
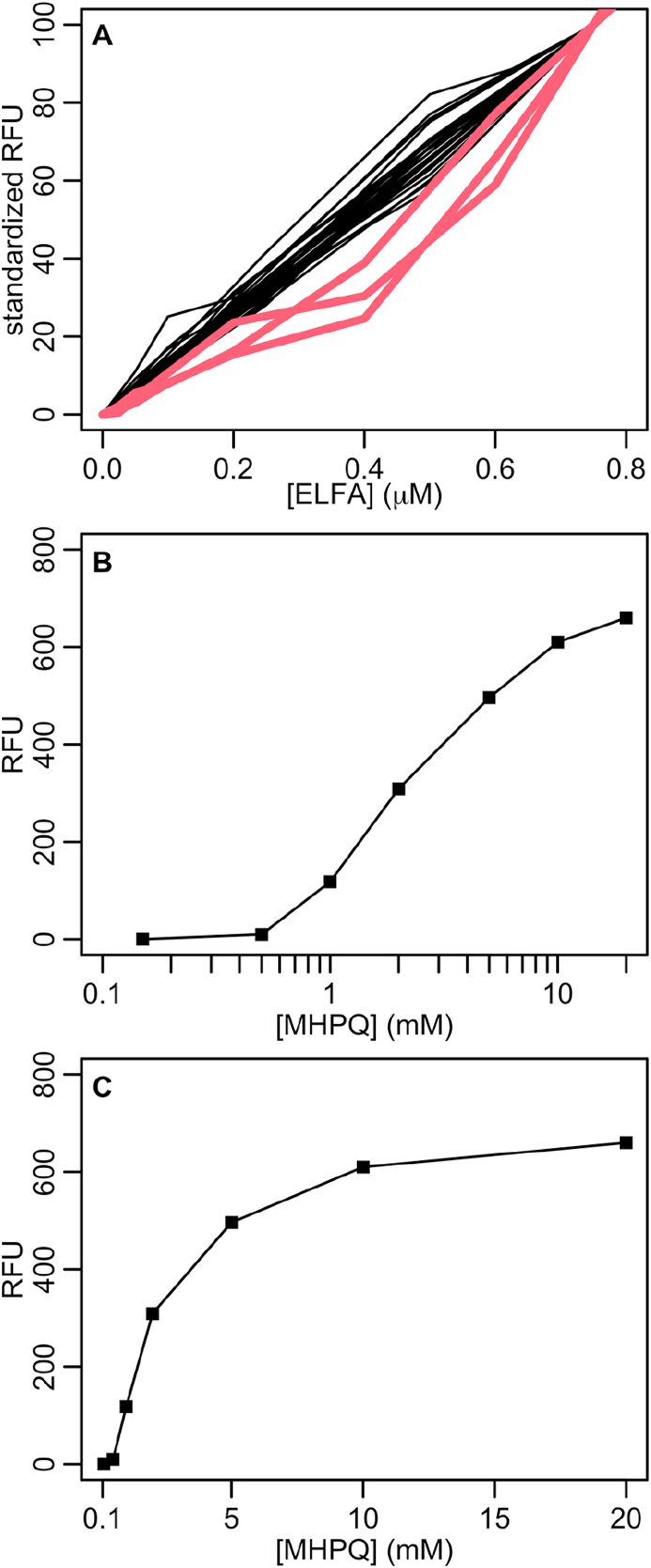
Fluorochrome concentration to fluorescence relationship. A: Comparison of 26 commercial ELFA calibration curves measured in spectrofluorimeter (thin black lines) with 3 self-made ELFA standards measured in a microplate reader (red thick lines). All measurements in each ELFA calibration curve were subtracted the fluorescence at 0 concentration (blank) and converted by the adequate conversion factor so that all 0.75 μM ELFA measurements were converted into 100. B: MHPQ concentration to fluorescence relationship as shown by [10]. C: the same in lineal scale, showing the lineal relationship range. Data shown in Figure 4 is available at Table S4.

There are two aspects worth to comment on in this protocol: the breakpoint in ELFA concentration to fluorescence relationship around 0.5 μM ELFA (Figure 3 B, C and D), and the accuracy of the ELFA concentration estimate. The 0.5 μM ELFA breakpoint in the dilution series of hydrolysed ELFA solutions (Figure 3 D) made us suspect that the ELFA standard-to-be solutions might have had ELFA concentrations much lower than expected. We used that hypothesis because it was conceivable that the ELFA solution manipulation (drying, volume transfer between wells and Eppendorf tubes, and dissolution in DMSO) had significantly diminished ELFA concentration and/or fluorescence. Moreover, according to the literature and our previous experience using ELFA, the lower limit of linear relationship between ELFA concentration and fluorescence should be below 0.5 μM ELFA. Old ELFA calibration curves measured with a spectrofluorimeter showed the lower limit of linear relationship to be much lower than 0.5 μM ELFA (Figure 4 A). Approximate but helpful information on this specific aspect was also found in the literature. A fluorochrome closely related to ELFA, 2-(5′-methoxy-2′-hydroxyphenyl)-4(3H)-quinazolinone (MHPQ), was shown to keep fluorescence to concentration linearity only for the concentration range (0.5–2 mM) [10] (Figure 4 B and C). MHPQ showed a 10% of maximum fluorescence at 0.8 mM, whereas ELFA had it at 0.1 mM. Also ELFA solubility limit was found to be at 0.1 mM [11]. Therefore, the ELFA loss of linearity might be somewhere below 0.1 mM but we cannot know where exactly according to the literature. Nevertheless, that hypothesis was rejected because ELFP incubations with phosphatase at the same concentration range showed barely the same breakpoint around 0.4 μM (Figure 3 B and C). In this case, no ELFA fluorescence or concentration loss can be attributed to any ELFA solution manipulation. Inversely, the breakpoint in Figure 3 B and C could be attributed to inefficient transformation of ELFP into ELFA at least in our low concentration reactions, but if that was the case the dilution series of already formed standard-to-be ELFA solutions should not show such a breakpoint and they do (Figure 3 D). In conclusion, the 0.4–0.5 μM ELFA breakpoint was likely due to linearity loss of the microwell reader as compared to the spectrofluorimeter at low fluorescence values, rather than an expression of much lower ELFA concentrations/fluorescence than expected.

The second aspect is the accuracy and correctness of the estimate of the ELFA concentration in the standard-to-be solutions. Although ELFA solutions 2 and 3 had similar and 4.5 times higher concentrations than ELFA solution 1 irrespective of incubation time, the absolute values were doubled from 463 min compared to 49 min incubation time (Table 1). Thus, two obvious questions arised: why is there such a difference, and what are the correct values?

Fading of already formed ELFA and slow hydrolysis of low concentration ELFP occurred during our experiment. By comparing Figure 3 B and C, we can notice the maximum value decreases along incubation time, indicating that the long incubation time might have caused ELFA to partially fade. On the other hand, initial ELFP concentration 0.4 μM increases a little bit only at the end of the incubation and overpasses the value of 200 RFU (Figure 3 B and C, green dot). This latter effect can also be observed in Figure 3A where the lower the ELFP substrate concentration, the later its maximum fluorescence peak occurs: the black 1 μM maximum occurs at min 35, the red 0.8 μM maximum occurs at min 49, and the blue 0.6 μM and green 0.4 μM maximums, at min 343. To minimise the interference of these two phenomena in our estimates, we took the maximum fluorescence records for each concentration regardless of incubation time (Figure 3 A). The resulting values (96, 449 and 336 μM ELFA) would be then our best estimates. An additional constraint supports the idea that at least min 436 estimations should be discarded. The constraint consisted in tracing dilutions from initial 1.5 mM ELFA to the obtainment of ELFA solutions 2 and 3, which allowed us to assign them provisional maximum values. Their actual values can only be equal to these provisional values or lower in case of ELFA molecules or fluorescence loss along their manipulation. In other words, observations are expected to be under the 1:1 dashed line (Figure 3 E and F). Any estimate higher than these maximum provisional values should be rejected as it is the case of ELFA solution 2 in min 436 (638 > 453 μM ELFA, Figure 3 F green line). In the end, these slow ELFP hydrolysis and fading issues should be bypassed using a higher enzyme concentration than we did and/or skipping fluorescence measurement to avoid exposition to light and fading. That is considered in the conclusion protocol below. Finally, although ELFA solution 2 provisional concentration approximately matched actual concentration, the provisional ELFA concentrations were clearly overestimated in ELFA solutions 1 and 3 (Table 1). This shows that evaporation, crystal formation, volume manipulation and fluorescence fading hamper any accurate direct determination of highly concentrated ELFA solutions concentration. Indeed, the present conversion using multiple reactions of low ELFP concentrations and ELFA dilution series can circumvent such limitation and it is accordingly included in the conclusion protocol, too.

To sum up, we run phosphatase reactions on ELFP substrate under the same conditions that triggered a complete hydrolysis of MUFP into MUF. We then evaporated resulting ELFA solution and dissolved it again in DMSO for storage. Finally, we determined the ELFA concentration of the solution in DMSO using a series of dilution factors and measuring it along with new phosphate reactions on low concentration ELFP. Overall it constitutes a DIY simple alternative, accessible to any environmental enzyme activity laboratory, to build-up their own ELFA standards necessary for SCPA quantification. The current protocol restores the possibility to quantify SCPA in aquatic microbes, to assess biogeochemically meaningful rates and enzyme kinetics properties, to combine quantitative SCPA analysis with other single-cell techniques, and to quantitatively compare new single-cell enzyme substrates to the well-established ELF technique.

## 4 Conclusions

### 4.1 Protocol to prepare ELFA standard solution

Reagents:

Step-by-step preparation:

1. *Reaction of ELFP with phosphatase.* Mix the vortexed reagents listed in Table 2 in a microwell of a 96 microwell plate. Let the reaction run in the dark, at +37 °C long enough so that the enzymatic reaction finished. Preferably, monitor it using a microwell plate reader. In this case, let the reaction run at least 1–1.5 h beyond the moment when you observe the ELFA fluorescence plateau, as ELFA production will still go on. Protect the microwell plate with a lid when not monitoring for increase of fluorescence. If you decide not to monitor the reaction, to be safe, run it for 6 h in the Eppendorf with screw cap where you will finally store the ELFA product solution. Final volume will be under the initial 200 μl due to evaporation (e.g. 140–170 μl).

**Table 2:** Reagent mix for phosphatase reaction on ELFP substrate to produce ELFA. Note 5 μl alkaline phosphatase is recommended instead of the 1 μl we used in this paper, in order to avoid excessive long incubation time and fluorescence fading.

Reagent	Volume	[Stock]	Final concentration	pH
ELFP	40 μl	5 mM ELFP	**1.0 mM**	Non specified (neutral)
		2 mM azide	0.4 mM	
AlkPase	5 μl	49098 units/ml	**1227.45 units/ml**	7.6
		3 M NaCl	75 mM NaCl	
		5 mM MgCl_2_	125 μM MgCl_2_	
		0.2 mM ZnCl_2_	5 μM ZnCl_2_	
		30 mM triethanolamine	0.75 mM triethanolamine	
Tris	20 μl	0.1M Tris	**0.01 M Tris**	7.9
MgCl_2_	20 μl	100 mM	**10 mM**	
MQ water	115 μl			
**TOTAL:**	**200 μl**			7.9

Bold refer to the main components in the dilution.
2. *Evaporation.* Add 100 μl 70% ethanol and mix thoroughly pipetting up and down or vortexing if you work the solution in a microwell or an Eppendorf tube, respectively. This will speed evaporation up. As a side-effect, most ELFA crystals will also dissolve, at least partially.
3. Add as much DMSO as possible (20–70 μl) with the pipette tip directly on the bottom of the microwell/Eppendorf tube and make sure to note down the volume for later concentration calculations. Use double gloves, lab coat and glasses when manipulating DMSO and work preferably under the fume hood. Be careful not to mix DMSO with the ELFA solution in ethanol. This operation might trigger some crystal formation but it will prevent excessive ELFA crystal formation during the evaporation step and facilitate posterior re-dissolution.
4. Let the solution dry in an oven at +37 °C over night, covered with a lid/cap to prevent any dust to fall in the well/Eppendorf. The next morning you should have only the DMSO volume and almost no crystals should be visible. (This evaporation step equalizes variability in evaporated volume during reaction and is necessary to move from aqueous solution to an adequate solvent like DMSO.)
5. *Re-dissolution.* Add as much DMSO as needed (130–180 μl) to attain a total volume comparable to the original one (200 μl). Pipette the volume up and down more than 100 times to the different cardinal directions to dissolve as much as possible any eventual ELFA crystals. Do it gently to prevent any splatters. If you ran the ELFP hydrolysis reaction in an Eppendorf tube instead of the microwell plate, you can vortex instead of pipetting up and down.
6. Transfer the volume to a screw cap Eppendorf tube and keep it hermetically closed in the dark, at +4 °C. This is the standard-to-be ELFA solution that will be used as a standard after the determination of its ELFA concentration. The high DMSO boiling point (+189 °C) makes it convenient to minimise evaporation and preserve volume and therefore ELFA concentration. You can alternatively use a common Eppendorf tube and seal it with Parafilm.
7. A provisional upward concentration of 1 mM ELFA will be assigned to this standard-to-be ELFA solution in DMSO, the solution whose actual ELFA concentration is to be determined. Some ELFA remains might be visible at the empty microwell, so the actual ELFA concentration is expected to be lower than the original 1 mM ELFP concentration.
8. *Determination of ELFA concentration.* Prepare a microplate with triplicates of a dilution series of the standard-to-be ELFA solution (e.g. 0, 0.4, 0.6, 0.8, 1, 1.2, 1.4, 1.6, 1.8, 2 μM ELFA) using MQ water and pH 7.9 0.01 mM f.c. Tris buffer as diluting agent and triplicate individual ELFP reactions at each of these concentrations, prepared in the same way as the one in step 1. Let the reaction run as in step 1, and monitor it until plateau using the microwell reader. When we used 1 μl alkaline phosphatase (254.5 units·ml^−1^ f.c.) three hours used to be enough to reach plateau although we monitored up to 7 h (e.g. at min 0, 30, 60, 90, 120, 180, 240, 300, 360, 420). Instead, use 5 times more enzyme to accelerate the reaction step and reduce ELFA fading.
9. Adjust a fluorescence to initial ELFP concentration regression line for the lineal relationship range (excluding at least 0 μM point). Use the maximum triplicate average fluorescence values obtained in each initial ELFP concentration, regardless of the reaction time point. This initial ELFP concentration will be considered equal to actual ELFA concentration, assuming a 100% efficiency of the reaction.
10. Use this regression line to convert the standard-to-be ELFA triplicate dilution series fluorescence values into actual ELFA concentrations. Adjust a second regression line between these estimated ELFA concentrations and their corresponding provisional ELFA concentrations, also for the lineal relationship range.
11. Use this second regression function to correct the provisionally assigned 1 mM concentration of our standard-to-be ELFA solution. This stable ELFA solution dissolved in a non-volatile solvent, and with known ELFA concentration is the self-made ELFA standard.

## Declarations

### Author contribution statement

Daniel Diaz-de-Quijano: Conceived and designed the experiments; Performed the experiments; Analyzed and interpreted the data; Contributed reagents, materials, analysis tools or data; Wrote the paper.

Cleo N. Stratman: Performed the experiments; Contributed reagents, materials, analysis tools or data; Wrote the paper.

Stella A. Berger: Contributed reagents, materials, analysis tools or data; Wrote the paper.

### Funding statement

Daniel Diaz-de-Quijano was supported by the Russian Foundation for Basic Research (RFBR, project number 20-04-00960), the Ministry of Science and Higher Education of the Russian Federation (Postdoctoral Program Project “5–100” and project number FSRZ-2020-0014), and the Leibniz-Institute of Freshwater Ecology and Inland Fisheries (IGB) travel grant.

Cleo N Stratman was supported by the Leibniz-Institute of Freshwater Ecology and Inland Fisheries (IGB) startup grant of Stella A Berger.

### Data availability statement

Data included in supplementary material and referenced in text.

### Declaration of interests statement

The authors declare no conflict of interest.

### Additional information

No additional information is available for this paper.

## Appendix A Supplementary data

ELFA_protocol_data_set-3.xlsx ELFA_protocol_data_set-3.xlsx

## References

[1] Jamet D., Aleya L., Devaux J. (1995). Diel changes in the alkaline phosphatase activity of bacteria and phytoplankton in the hypereutrophic Villerest reservoir (Roanne, France). Hydrobiologia.

[2] Kang W., Wang Z.H., Liu L., Guo X. (2019). Alkaline phosphatase activity in the phosphorus-limited southern Chinese coastal waters. J. Environ. Sci. (China).

[3] Healey F.P., Hendzel L.L. (1980). Physiological indicators of nutrient deficiency in lake phytoplankton. Can. J. Fish. Aquat. Sci. Can. Des Sci. Halieutiques Aquat. Ottawa..

[4] Cembella A.D., Antia N.J., Harrison P.J. (1984). The utilization of organic and inorganic phosphorus compounds as nutrients by eukaryotic microalgae- A multidisciplinary perspective: Part 1. Crit. Rev. Microbiol..

[5] Jansson M., Olsson H., Pettersson K. (1988). Phosphatases; origin, characteristics and function in lakes. Hydrobiologia.

[6] Hoppe H.G. (2003). Phosphatase activity in the sea. Hydrobiologia.

[7] Tanaka T., Henriksen P., Lignell R., Olli K., Seppälä J., Tamminen T., Thingstad T.F. (2006). Specific affinity for phosphate uptake and specific alkaline phosphatase activity as diagnostic tools for detecting phosphorus-limited phytoplankton and bacteria. Estuar. Coast.

[8] Litchman E., Klausmeier C.A. (2008). Trait-based community ecology of phytoplankton. Annu. Rev. Ecol. Evol. Syst..

[9] Berman-Frank I., Quigg A., Finkel Z.V., Irwin A.J., Haramaty L. (2007). Nitrogen-fixation strategies and Fe requirements in cyanobacteria. Limnol. Oceanogr..

[10] Huang Z., Terpetschnig E., You W., Haugland R.P. (1992). 2-(2’-Phosphoryloxyphenyl)-4(3H)-quinazolinone derivatives as fluorogenic precipitating substrates of phosphatases. Anal. Biochem..

[11] Huang Z., You W., Haugland R.P., Paragas V.B., Olson N.A. (1993). A novel fluorogenic substrate for detecting alkaline phosphatase activity in situ. J. Histochem. Cytochem..

[12] Paragas V.B., Kramer J.A., Fox C., Haugland R.P., Singer V.L. (2002). The ELFR-97 phosphatase substrate provides a sensitive, photostable method for labelling cytological targets. J. Microsc..

[13] Paragas V.B., Zhang Y.Z., Haugland R.P., Singer V.L. (1997). The ELF-97 alkaline phosphatase substrate provides a bright, photostable, fluorescent signal amplification method for FISH. J. Histochem. Cytochem..

[14] Larison K.D., BreMiller R., Wells K.S., Clements I., Haugland R.P. (1995). Use of a new fluorogenic phosphatase substrate in immunohistochemical applications. J. Histochem. Cytochem..

[15] González-Gil S., Keafer B.A., Jovine R.V.M., Aguilera A., Lu S., Anderson D.M. (1998). Detection and quantification of alkaline phosphatase in single cells of phosphorus-starved marine phytoplankton. Mar. Ecol. Prog. Ser..

[16] Štrojsová A., Vrba J., Nedoma J., Komárková J., Znachor P. (2003). Seasonal study of extracellular phosphatase expression in the phytoplankton of a eutrophic reservoir. Eur. J. Phycol..

[17] van Ommen Kloeke F., Geesey G.G. (1999). Localization and identification of populations of phosphatase-active bacterial cells associated with activated sludge flocs. Microb. Ecol..

[18] van Ommen Kloeke F., Baty A.M., Eastburn C.C., Diwu Z., Geesey G.G. (1999). Novel method for screening bacterial colonies for phosphatase activity. J. Microbiol. Methods.

[19] Nedoma J., Vrba J. (2006). Specific activity of cell-surface acid phosphatase in different bacterioplankton morphotypes in an acidified mountain lake. Environ. Microbiol..

[20] Stibal M., Anesio A.M., Blues C.J.D., Tranter M. (2009). Phosphatase activity and organic phosphorus turnover on a high Arctic glacier. Biogeosciences.

[21] Carlsson P., Caron D.A. (2001). Seasonal variation of phosphorus limitation of bacterial growth in a small lake. Limnol. Oceanogr..

[22] Van Wambeke F., Nedoma J., Duhamel S., Lebaron P. (2008). Alkaline phosphatase activity of marine bacteria studied with ELF 97 substrate: success and limits in the P-limited Mediterranean Sea. Aquat. Microb. Ecol..

[23] Cao X., Štrojsová A., Znachor P., Zapomělová E., Liu G., Vrba J., Zhou Y. (2005). Detection of extracellular phosphatases in natural spring phytoplankton of a shallow eutrophic lake (Donghu, China). Eur. J. Phycol..

[24] Cao X., Song C., Zhou Y., Štrojsová A., Znachor P., Zapomělová E., Vrba J. (2009). Extracellular phosphatases produced by phytoplankton and other sources in shallow eutrophic lakes (Wuhan, China): taxon-specific versus bulk activity. Limnology.

[25] Cao X., Song C., Zhou Y. (2010). Limitations of using extracellular alkaline phosphatase activities as a general indicator for describing P deficiency of phytoplankton in Chinese shallow lakes. J. Appl. Phycol..

[26] Štrojsová M., Vrba J. (2005). Direct detection of digestive enzymes in planktonic rotifers using enzyme-labelled fluorescence (ELF). Mar. Freshw. Res..

[27] Rengefors K., Pettersson K., Blenckner T., Anderson D.M. (2001). Species-specific alkaline phosphatase activity in freshwater spring phytoplankton: application of a novel method. J. Plankton Res..

[28] Rengefors K., Ruttenberg K.C., Haupert C.L., Taylor C., Howes B.L., Anderson D.M. (2003). Experimental investigation of taxon-specific response of alkaline phosphatase activity in natural freshwater phytoplankton. Limnol. Oceanogr..

[29] Dignum M., Hoogveld H.L., Matthijs H.C.P., Laanbroek H.J., Pel R. (2004). Detecting the phosphate status of phytoplankton by enzyme-labelled fluorescence and flow cytometry. FEMS Microbiol. Ecol..

[30] Dignum M., Hoogveld H.L., Floris V., Gons H.J., Matthijs H.C.P., Pel R. (2004). Flow cytometric detection of phosphatase activity combined with C-13-CO2 tracer-based growth rate assessment in phytoplankton populations from a shallow lake. Aquat. Microb. Ecol..

[31] Duhamel S., Gregori G., Van Wambeke F., Nedoma J. (2009). Detection of extracellular phosphatase activity at the single-cell level by enzyme-labeled fluorescence and flow cytometry: the importance of time kinetics in ELFA labeling. Cytom. Part A..

[32] Diaz-de-Quijano D., Horňák K., Palacios P., Felip M. (2014). 3D restoration microscopy improves quantification of enzyme-labelled fluorescence (ELF)-based single-cell phosphatase activity in plankton. Cytom. Part A..

[33] Vrba J., Macholdová M., Nedbalová L., Nedoma J., Šorf M. (2018). An experimental insight into extracellular phosphatases – differential induction of cell-specific activity in green algae cultured under various phosphorus conditions. Front. Microbiol..

[34] Nedoma J., Štrojsová A., Vrba J., Komárková J., Šimek K. (2003). Extracellular phosphatase activity of natural plankton studied with ELF97 phosphate: fluorescence quantification and labelling kinetics. Environ. Microbiol..

[35] Johnson I., Spence M. (2010). Chapter 10 - enzyme substrates and assays. Mol. Probes ® Handbook. A Guid. to Fluoresc. Probes.

[36] Zhou M., Upson R.H., Diwu Z., Haugland R.P. (1996). A fluorogenic substrate for [beta]-glucuronidase: applications in fluorometric, polyacrylamide gel and histochemical assays. J. Biochem. Biophys. Methods.

[37] Diwu Z., Lu Y., Upson R.H., Zhou M., Klaubert D.H., Haugland R.P. (1997). Fluorescent molecular probes I. The synthesis and biological properties of an ELF® β-glucuronidase substrate that yields fluorescent precipitates at the enzymatic activity sites. Tetrahedron.

[38] Baty A.M., Eastburn C.C., Diwu Z., Techkarnjanaruk S., Goodman A.E., Geesey G.G. (2000). Differentiation of chitinase-active and non-chitinase-active subpopulations of a marine bacterium during chitin degradation. Appl. Environ. Microbiol..

[39] Baty A.M., Diwu Z., Dunham G., Eastburn C.C., Geesey G.G., Goodman a E., a Suci P., Techkarnjanaruk S. (2001). Characterization of extracellular chitinolytic activity in biofilms. Methods Enzymol..

[40] Štrojsová A., Dyhrman S.T. (2008). Cell-specific beta-N-acetylglucosaminidase activity in cultures and field populations of eukaryotic marine phytoplankton. FEMS Microbiol. Ecol..

[41] Novotná J., Nedbalová L., Kopáček J., Vrba J. (2010). Cell-specific extracellular phosphatase activity of Dinoflagellate populations in acidified mountain lakes. J. Phycol..

[42] Takahashi T., Otsubo T., Ikeda K., Minami A., Suzuki T. (2014). Histochemical imaging of alkaline phosphatase using a novel fluorescent substrate. Biol. Pharm. Bull..

[43] Polaske N.W., Kelly B.D., Ashworth-Sharpe J., Bieniarz C. (2016). Quinone methide signal amplification: covalent reporter labeling of cancer epitopes using alkaline phosphatase substrates. Bioconjug. Chem..

[44] Takahashi T., Takano M., Kurebayashi Y., Agarikuchi T., Suzuki C., Fukushima K., Takahashi S., Otsubo T., Ikeda K., Minami A., Suzuki T. (2015). Rapid fluorescent detection assay for human parainfluenza viruses. Biol. Pharm. Bull..

[45] Díaz-de-Quijano D., Felip M. (2011). A comparative study of fluorescence-labelled enzyme activity methods for assaying phosphatase activity in phytoplankton. A possible bias in the enzymatic pathway estimations. J. Microbiol. Methods.

[46] Haugland R.P., Johnson I.D. (1993). Detecting enzymes in living cells using fluorogenic substrates. J. Fluoresc..

[47] R Development Core Team (2017). R: a language and environment for statistical computing. R Found. Stat. Comput..

